# Transcriptional signature of accessory cells in the lateral line, using the *Tnk1bp1*:EGFP transgenic zebrafish line

**DOI:** 10.1186/1471-213X-12-6

**Published:** 2012-01-24

**Authors:** Martine Behra, Viviana E Gallardo, John Bradsher, Aranza Torrado, Abdel Elkahloun, Jennifer Idol, Jessica Sheehy, Seth Zonies, Lisha Xu, Kenna M Shaw, Chie Satou, Shin-ichi Higashijima, Brant M Weinstein, Shawn M Burgess

**Affiliations:** 1 National Human Genome Research Institute, National Institutes of Health, Bethesda, MD 20892, USA; 2 National Institute of Child Health and Human Development, Bethesda, MD 20892, USA; 3 Department of Anatomy and Neurobiology, Medical school, University of Puerto Rico, PR 00936, USA; 4 National Institutes of Natural Sciences, Okazaki Institute for Integrative Biology, Okazaki, Aichi 444-8787 Japan; 5 National Cancer Institute, Bethesda, MD 20892, USA

**Keywords:** Regeneration, hair cells, progenitor cells, lateral line, zebrafish, supporting cells, accessory cells, microarrays, Tnk1bp1

## Abstract

**Background:**

Because of the structural and molecular similarities between the two systems, the lateral line, a fish and amphibian specific sensory organ, has been widely used in zebrafish as a model to study the development/biology of neuroepithelia of the inner ear. Both organs have hair cells, which are the mechanoreceptor cells, and supporting cells providing other functions to the epithelium. In most vertebrates (excluding mammals), supporting cells comprise a pool of progenitors that replace damaged or dead hair cells. However, the lack of regenerative capacity in mammals is the single leading cause for acquired hearing disorders in humans.

**Results:**

In an effort to understand the regenerative process of hair cells in fish, we characterized and cloned an *egfp *transgenic stable fish line that trapped *tnks1bp1*, a highly conserved gene that has been implicated in the maintenance of telomeres' length. We then used this Tg(*tnks1bp1*:EGFP) line in a FACsorting strategy combined with microarrays to identify new molecular markers for supporting cells.

**Conclusions:**

We present a Tg(*tnks1bp1*:EGFP) stable transgenic line, which we used to establish a transcriptional profile of supporting cells in the zebrafish lateral line. Therefore we are providing a new set of markers specific for supporting cells as well as candidates for functional analysis of this important cell type. This will prove to be a valuable tool for the study of regeneration in the lateral line of zebrafish in particular and for regeneration of neuroepithelia in general.

## Background

The field of auditory biology has made tremendous strides over recent decades, but molecular characterization has been greatly hampered by the paucity of available neuroepithelia and the difficulty in accessing the inner ear. In mammals, the sensory tissue is deeply buried in the skull and presents few and small discrete sensory regions.

The lateral line, a sensory organ specific to fish and amphibians, offers an excellent alternative "model organ" for the inner ear, because of its strong similarities and common developmental program [1-4]. The superficially and stereotypically distributed sensory patches along the side of the fish are called neuromasts [3,5]. Like neuroepithelia in the inner ear, neuromasts are composed of two main cell types, hair cells and supporting cells. Hair cells are mechanoreceptors, which are transducing the mechanical deflection of their apical cilia into electrical signals that are relayed to the CNS [6]. The lateral line is directly exposed to its aqueous surroundings and the hair cells are triggered by water movements, influencing swimming behaviors [7]. The supporting cells are still a poorly defined and described group of cells. They comprise at least two different cell types, which have been distinguished mainly on morphological criteria, called the supporting (or support) cells and the mantle cells [8]. We will refer to them in aggregate as accessory cells. They have a structural and cohesive role in neuroepithelia, but more remarkably, in non-mammalian vertebrates, they comprise the progenitor pool that replaces damaged or destroyed hair cells throughout the life of the animal [9-12]. The regenerative property of supporting cells is lost in mammals after birth, therefore rendering the absence/damage of hair cells irreversible. Because zebrafish are able to regenerate lost hair cells in both the ear and in the lateral line, the neuromasts offers an attractive *in vivo *system to study the development and the regeneration of the sensory neuroepithelia [13-15].

Whereas hair cells have been extensively studied and characterized, offering a large panel of vital stains and molecular markers, very few markers are available for accessory cells resulting in a challenge for the field. We identified a "gene-trap" transgenic line that expresses GFP in accessory cells of the lateral line and of the olfactory sensory epithelium. We show that the gene-trap construct landed in a gene we have characterized as being the homolog of tankyrase 1 binding protein 1 (*tnks1bp1*), a gene interacting with tankyrase 1, which has several putative functions in cells including telomere elongation [16]. Additionally, we used the transgenic line as a tool for enriching accessory cells and defining their transcriptional signature. First, we FAC sorted homogenates of Tg(*tnks1bp1*:EGFP) larvae to isolate GFP positive cells. We extracted their RNA and hybridized it against reference RNA made from non-fluorescent cells. Genes that determined as up-regulated or enriched were specific to accessory cells and essentially provide a transcriptional signature. We present here the transcriptional profiling, providing a new set of markers specific for accessory cells of the lateral line. This tool will be valuable for studying regeneration in the lateral line in particular and regeneration of neuroepithelia in general.

## Results

### The transgenic line Tg(*tnks1bp1*:EGFP) expressed EGFP specifically in the accessory cells of the lateral line and of the olfactory epithelium

**Figure 1:**
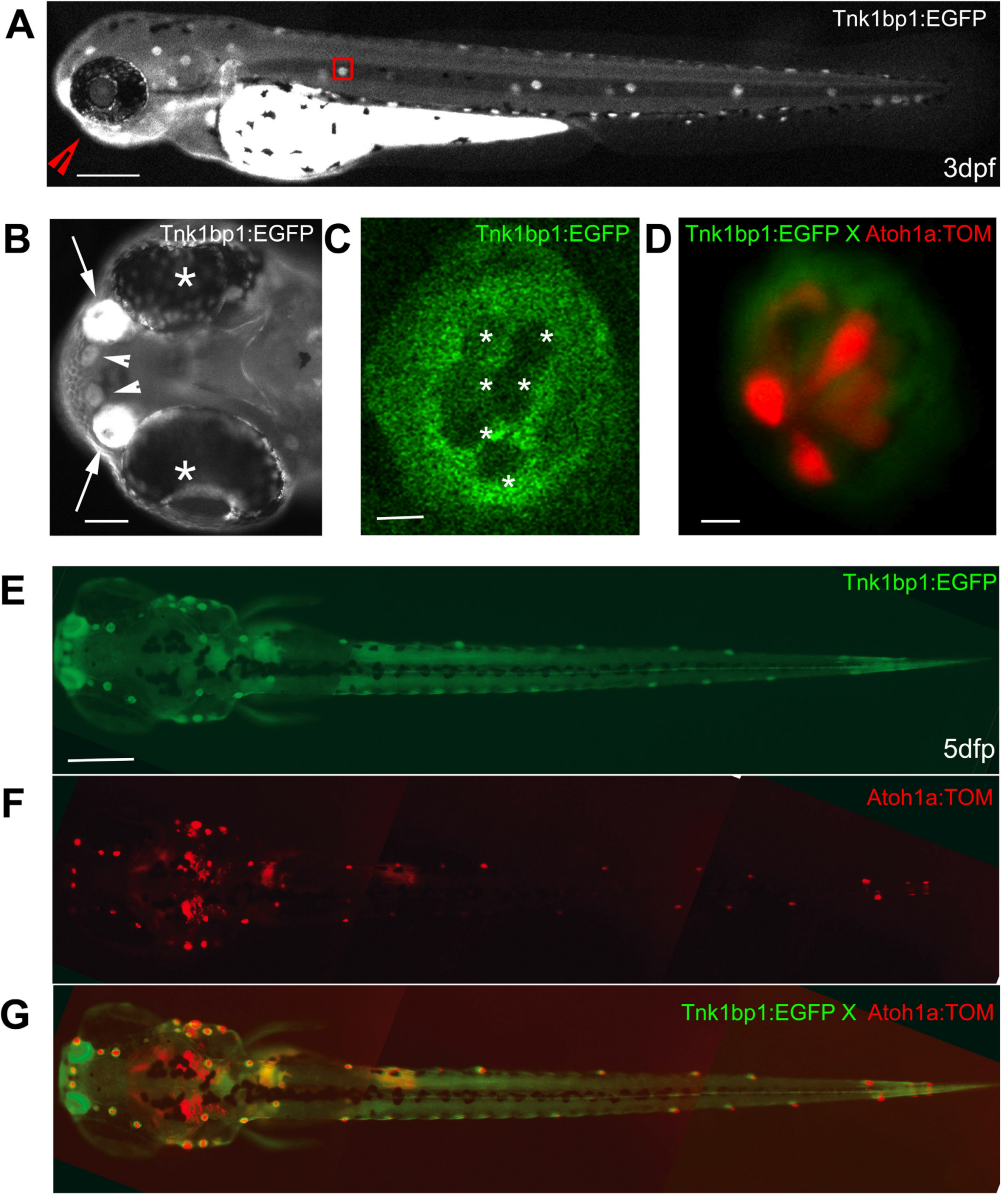
**Live imaging of a 3 day old Tg (*tnks1bp1*:EGFP) embryo and a 5 day old Tg(*tnks1bp1*:EGFP) *x *Tg (*atoh1a*:dTOM) double transgenic larva**. **A**. GFP is expressed in all of the neuromasts in the anterior (head) and posterior (trunk and tail) lateral line, as shown in a three day old embryo. **B**. Ventral view (red arrowhead in A) of the rostral head region of the embryo, showing a strong GFP expression in the olfactory epithelia (white arrows) between the eyes (white asterisks) and in the two more rostral neuromasts (white arrowheads). **C**. Magnification of a neuromast (red box in A) in a 3 dpf live embryo, showing GFP expression excluded from the six centrally located hair cells (white asterisks). **D**. Neuromast of a Tg(*tnks1bp1*:EGFP) × Tg(*atoh1a*:dTOM) 5 dpf double transgenic animal expressing GFP (green) in the accessory cells and TOMATO (red) in hair cells. **E, F and G**. Dorsal view of a 5 day dpf Tg(*tnks1bp*:GFP) × Tg(*atoh1a*:dTOM) double transgenic larva. - 200 microns in A and E, 40 microns in B, and 5 microns in C and D.

We focused our attention on one particular transgenic line found in a gene trap effort, which had a highly restricted expression pattern prominent in the supporting cells and mantle cells. Starting at 2 days post fertilization (dpf) the GFP was limited to two obvious developing sensory organs, the lateral line (Figure 1A lateral view of a 3 dpf whole embryo and arrowheads in Figure 1B, E and 1G dorsal view of a 5 dpf larva) and the olfactory epithelium (Figure 1B, arrows in a ventral view of the head, as seen from the red arrow head in Figure 1A). The signal from the yolk sac and extension was due to auto-fluorescence. The GFP expression was persistent in adult animals (not shown). When taking a closer look at one of the functional units of the lateral line, called the neuromast, (red square in Figure 1A and close up in Figure 1C) we noticed that the whole sensory structure, with the exception of the centrally located cells (white stars in Figure 1C), was expressing GFP. Neuromasts have been well characterized and described previously as centrally located mechanoreceptors, also called hair cells surrounded by supporting cells [17].

**Figure 2:**
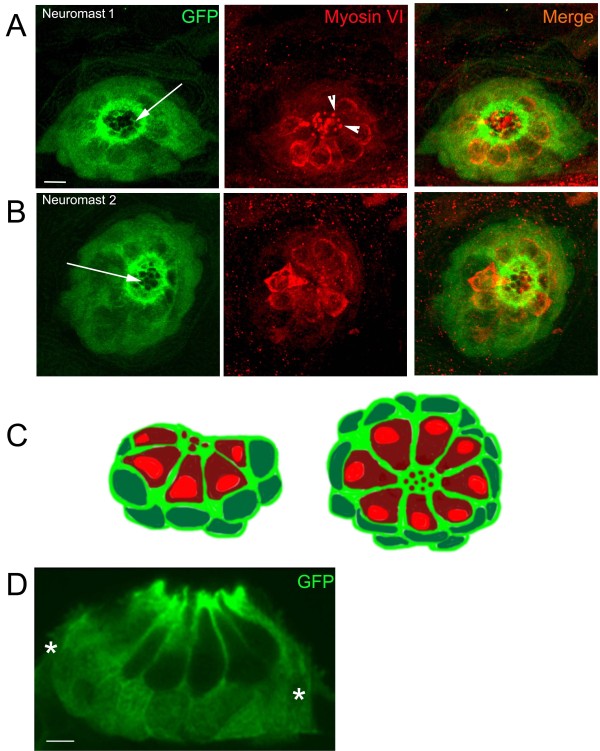
**Immuno-staining in neuromasts of 5 dpf Tg (*tnks1bp1*:EGFP) larvae**. **A. B**. Confocal images of two different neuromasts (1 and 2) double-stained with anti-GFP (green, first and last columns) and anti-Myosin VI (red, second and last columns). The GFP positive cells (green) are the accessory cells (*i.e. *a combination of supporting cells and mantle cells). Around the apical pole of hair cells, accessory cells form a precisely organized honeycomb like annular structure (white arrows in A and B, first columns). Hair cells send their tightly packed hair bundles through the openings in the top, (white arrowheads in A, middle panel). **C**. Schematic cross-section (left image) and dorsal view (right image) of a neuromast illustrating the respective position of the accessory cells (cytoplasm light green and nucleus dark green) and the hair cells (cytoplasm dark red and nucleus light red). **D**. Cryosection of a neuromast, immunolabeled with an anti-GFP antibody. GFP expression is excluded from all hair cells (nucleus and cytoplasm) and found in all accessory cells, comprising the supporting and mantle cells (white stars). - 10 microns in A and B, 5 microns in D.

To confirm the identity of the GFP negative cells, we crossed the transgenic Tg(*tnks1bp1*:EGFP) line into another previously published transgenic line, Tg(*atoh1a*:dTomato), which expresses the Tomato reporter gene in hair cells [18] (Figure 1D, F and 1G). No overlapping EGFP and TOMATO expression was found, as illustrated in Figure 1D, EGFP (green) in accessory cells and TOMATO (red) in hair cells. Because some transgenic lines do not faithfully recapitulate all of the normal gene expression, to further confirm the identity of the GFP positive cells we counterstained fixed 5 dpf larvae with two antibodies, an anti-GFP (green in Figure 2A and 2B, left and right panels) and one to a well-documented hair cell specific marker: myosin VI (red in Figure 2A and 2B, middle and right panels) [19]. As seen in two different examples of Tg(*tnks1bp1*:EGFP) transgenic neuromasts (Figure 2A and 2B), the centrally located hair cells were GFP negative and Myosin VI positive. All accessory cells were expressing GFP. In the apical part of the neuromast, supporting cells were forming a "honey comb like" structure (white arrows in Figure 2A and 2B) through which hair cells were sending their hair bundles (arrowheads in Figure 2A). Each hair cell appeared as being isolated from its neighbor by a thin cytoplasmic furrow formed by the surrounding supporting cells (Figure 2C, Schematics of transverse (left) and dorsal (right) views of a neuromast). Hair cells were visible in dark red with light red nuclei and accessory cells in light green with dark green nuclei). To further assess that GFP was absent from hair cells, we performed cryosections followed by antibody staining in transgenic larvae. The GFP staining was completely excluded form hair cells and restricted to accessory cells (Figure 2D). The more peripheral mantle cells, which have been described previously [8] were also stained (white stars). We therefore concluded, that the Tg(*tnks1bp1*:EGFP) was the specific to accessory cells (supporting and mantle cells) in the lateral line.

### The Tol2 transposon construct traps the putative zebrafish homolog of *tnks1bp1*

**Figure 3:**
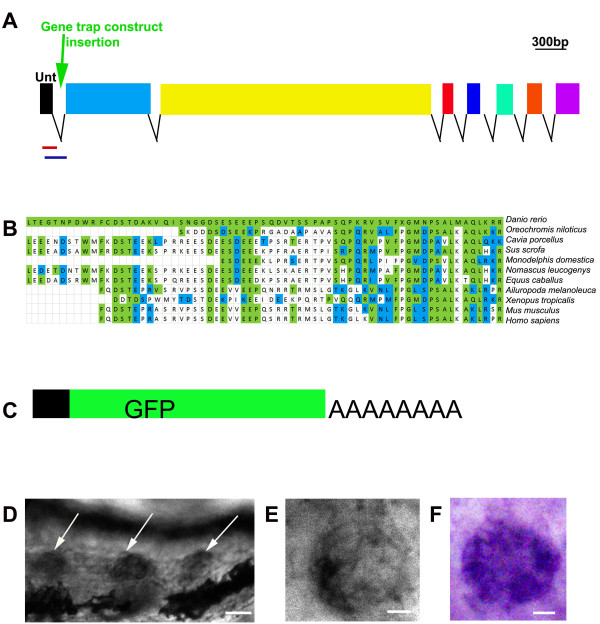
**The trapped gene is a homolog of the tnk1bp1 gene**. **A**. The *tnks1bp *gene. It encodes 8 exons with the first one (black square), being untranslated. The gene-trap construct landed in the first intron (green arrow), resulting in a product described in (C). Two sets of primers for qRT-PCR were designed to track the presence of alternatively spliced products in transgenic larvae. The amplified products are marked by the two colored lines under the gene depiction. The lines illustrate respectively: red for the product comprised of exon1 (black square) to the *gfp *from the gene-trap (not depicted), blue for exon1 (black square) to exon2 (blue square of the *tnks1bp1 *gene. **B**. Alignment of a stretch of ~70AA of a portion of the C-term of the putative TNKS1BP1 product with ten partial sequences of the putative product of the TNK1BP1 as sequenced/predicted from 10 species. The amino acids highlighted in green are identical and the ones in blue are conservatively substituted. **C**. Symbolic representation of the mRNA, which was found in abundance in the transgenic embryos. This messenger results from the fusion of the first untranslated exon 1 of the *tnks1bp1 *gene and the *gpf *sequence followed by a polyA tail, elements encoded by the integrated transposon construct (green arrow in A). **D**. Three tail neuromasts are positive (white arrows) by *in situ *hybridization with a probe against the *tnks1bp1 *gene. **D. and E**. Neuromasts after WISH (against *tnks1bp1*), showing stronger hybridization in the more external cells, corresponding to accessory cells.

The accessory cells have been described as comprising a pool of cells that can give rise to new hair cells after hair cell ablation [9,10,14]. In order to further characterize the accessory cells, we identified the gene trapped by the Tol2 construct in the Tg(*tnks1bp1*:EGFP) transgenic line. We mapped the genomic region adjacent to the transposon insertion site, using methods described previously [20]. We found the insertion in the first intron of a predicted gene that mapped to chromosome 5 (ENSDARG00000068760). We cloned the entire gene with its 5' and 3' UTR. The cDNA was 4628 nucleotides (nts) long comprised of 8 exons (Figure 3A). The first exon was short (132nts) and untranslated. In the second exon (852nts long) we found the translational start (in position 148 from the beginning of the cDNA). The third exon was remarkably long (2679 nts), followed by 5 short ones (98, 129, 165, 135 and 225 nts) (Figure 3A). The longest putative encoded protein would be 1396 amino acids (AA). The complete sequence has been submitted to GenBank [Genebank: JN106182.1].

Next, we searched the sequence databases for homologies and other available information. No conserved domains were found throughout the putative coding region. When performing BLAST on the NCBI website http://blast.ncbi.nlm.nih.gov/[21], we found a stretch of ~ 65AA encoded by part of exon 6, the whole exon 7 and part of exon 8 in the 3' end of the gene that showed significant identity with a region of the product of the tankyrase1 binding protein (TNKS1BP1). The degree of identity of this conserved stretch was varying from 35 to 50% in 15 different species, ranging from tilapia to human (Figure 3B, 11 species are shown). This gene was of particular interest as the *tankyrase1 *gene has been implicated in regulating the length of telomeres [22], a function that would be highly relevant to stem cell populations. In addition another stretch of ~40AA in the N-terminus of the putative protein was showing a significant identity (37%) with the predicted TNKS1BP1 protein in *Ailuropoda melanoleuca *(giant panda, not shown). Nearly all of the *tnks1bp1 *gene products in different species have been computer predicted only, and the lack of identity with the rest of the sequence does not exclude the possibility that this is not a precise homolog of *tnks1bp1*, but potentially a gene related to the tkns1bp1 gene with an expression pattern restricted to accessory cells of the lateral line. Therefore, we concluded that the trapped gene encodes a putative homolog of the *tnks1bp1 *gene.

To confirm that the insertion was indeed trapping the *tnks1bp1 *gene, we performed RT-PCR targeting a transcript that was a fusion product of *tnks1bp1 *and *egfp*. We found this product in abundance in the transgenic animals. Upon sequencing the transcript, we found it was the result of the fusion of the 5'UTR of *tnks1bp1 *(the first untranslated exon) with the *gfp *sequence followed by a polyA tail (Figure 3B). This was the expected result considering the construct used in the gene trap approach (see materials and methods). This confirmed that the construct had indeed landed in the *tnks1bp1 *gene and that the GFP expression was faithfully reporting the expression of the endogenous *tnks1bp1 *gene.

We performed whole mount *in situ *hybridization (WISH) in embryos of various stages using a probe designed against several regions of the *tnks1bp1 *gene. The WISH reproduced the GFP expression in all the neuromasts (white arrows pointing to the three tail neuromasts in Figure 3D) and in the olfactory epithelium (not shown). The staining was present in all of the accessory cells (as seen in two different neuromasts in Figure 3E and 3F). Taken together, these results clearly showed that the *tnks1bp1 *gene was expressed in all accessory cells of the lateral line and the olfactory epithelium as reported by the GFP insertion in the Tg(*tnks1bp1*:EGFP) transgenic line and in the WISH against *tnks1bp1*. This confirmed that *tnks1bp1 *is a highly specific accessory cell marker.

We checked for phenotypic differences in heterozygous and homozygous carriers of the transgene. Homozygotes were relatively easy to distinguish as the expression of GFP was noticeably stronger. The lateral line developed normally in all the larvae at all stages that we checked (not shown). Next, we looked if the regeneration of hair cells was affected in either heterozygous or homozygous carriers using an assay described previously [15]. Again we did not see a significant phenotype (data not shown). To be able to conclude on the absence of phenotype, we needed to determine if the insertion of the gene-trap was indeed completely disrupting the *tnks1bp1 *gene.

In order to assess the presence of various possible splicing products in wild-type and homozygous larva, we designed two different sets of primers for qRT-PCR on RNA extracted from wild-type (no GFP) and homozygous (strong GFP) larvae. Primer set one (blue line in Figure 3A) amplified from exon 1 to exon 2 identifying the wild-type message. Primer set two (red line in Figure 3A) amplified the fusion of exon 1 with the EGFP trap exon identifying "trapped" message. As expected the product from set 2 was absent in wild-type larva. The other primer showed no measurable significant difference between wild-type and homozygous transgenics (not shown). Therefore, we concluded that in Tg(*tnks1bp1*:EGFP) homozygote carriers, the gene trap was significantly spliced around, allowing for both the production of the wild-type and the gene-trap transcripts. Thus, the expression of the *tnks1bp1 *gene was not significantly disrupted in homozygous carriers.

Next, we used a knockdown approach where we injected two different morpholinos against the ATG of the *tnks1bp1 *gene in wild-type and Tg(*tnks1bp1*:EGFP) homozygote embryos. A mismatch control was injected in parallel. None of the morphants exhibited significant phenotypes (not shown). We concluded that (excluding poor efficacy of both of the designed morpholinos) the significant functions of the *tnks1bp1 *gene might come in play only after the time window during which the morpholinos have a reliable effect (about 3 dpf), or the gene is not essential for the functions we tested. This was compatible with a late onset of the *tnks1bp1 *gene expression around 2 dpf, as reported by the transgenic line. Alternative approaches will have to be pursued to further assess the role of the *tnks1bp1 *gene.

### Defining the transcriptome of accessory cells of the lateral line and the olfactory sensory epithelium

**Figure 4:**
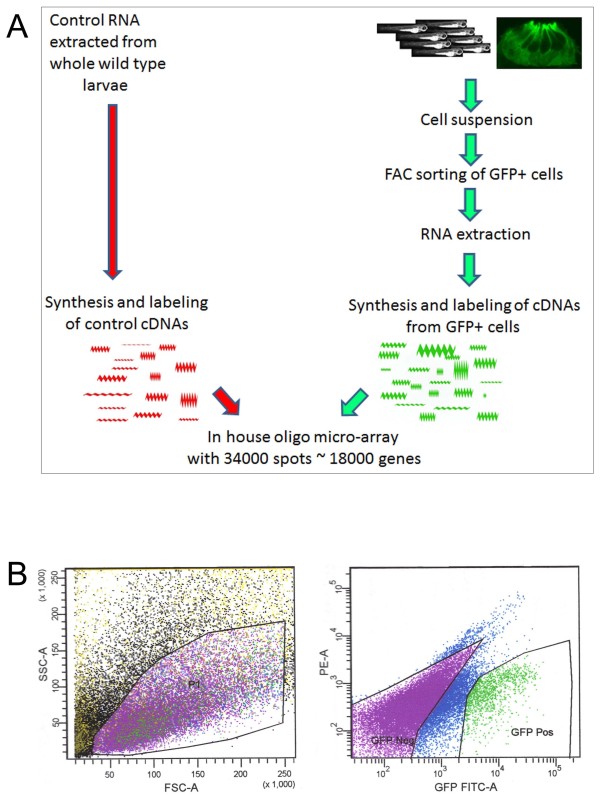
**Strategy to establish the transcriptional profile of accessory cells combining FACS and microarrays**. **A**. Schematic of the experimental procedures. **B**. illustration of the gating used for the FACS. The left plot (showing the P1 gate) was used to sort cells according to cell size (forward scatter, FSC-A) vs. granularity (side scatter, SSC-A). The right plot discriminated cells according to GFP fluorescence intensity (GFP FITC-A) vs. Phycoerythrin (PE-A). Gates were selected as shown to sort GFP negative (GFP Neg) and GPP positive (GFP Pos) cells.

Only a handful of specific markers are available for accessory cells in the neuromast of the lateral line. To address this need and in order to better characterize those cells at the molecular level, we decided to undertake a genomic approach using the Tg(*tnks1bp1*:EGFP) line. In a 5 dpf larva, we roughly estimated the number of accessory cells to be in the range of 1000-2000 cells. The larva has ~48 neuromasts in its lateral line with ≈30 accessory cells/neuromast + ≈ 2 × 100 cells/olfactory epithelium = 1650. This represented a small number of cells compared to the total number of cells in a larva at this stage, which in our estimation was in the range of several billion. As we wanted to address the composition of transcripts in the accessory cells specifically (transcriptome), we needed to isolate them from the remaining larval cells. The method we used is schematized in Figure 4A. Briefly, we collected 5 dpf Tg (*tnks1bp1*:EGFP) larvae and dissociated them to form a cell suspension. In the next step, we passed the cell suspension through fluorescence activated cell sorting (FACS) to separate GFP positive (GFP +) cells and collected this fraction. The settings for the sorting were carefully determined empirically and exactly reproduced in each subsequent experiment. An illustration of those settings is shown (Figure 4B, left and right panels). The left plot (showing the P1 gate) was used to sort cells according to cell size (forward scatter, FSC-A) vs. granularity (side scatter, SSC-A). The right plot discriminated cells, according to the GFP fluorescence intensity of cells (GFP FITC-A) vs. Phycoerythrin (PE-A). Gates were demarcated to sort GFP negative (GFP Neg, GFP-) and GFP positive (GFP Pos, GFP +) cells. Both of those fractions were visually assessed under the microscope for the absence or presence of GFP respectively (data not shown). The GFP + fraction, highly enriched in GFP positive cells, now theoretically contained mostly accessory cells.

Next, we extracted the RNA from the GFP + cells population using traditional phenol chloroform extraction methods. In parallel we prepared control RNA, which we extracted from whole wild-type larvae at different development stages. We synthesized and labeled cDNA probes from both RNA populations, which were then co-hybridized custom printed microarrays [23]. Each chip represented ≈34,000 spots, which corresponded to roughly 18,000 genes. mRNA of genes that were exclusively or overwhelmingly present in the accessory cell population would appear as up-regulated and we referred to these transcripts hereafter as, enriched.

### Analysis of the microarray data

The microarray experiment was performed with multiple biological replicates (n = 15) and technical replicates (dye swap, n = 5). After normalization (see material & methods), the results were analyzed in parallel using two separate microarray analysis resources, GeneSifter http://www.geospiza.com/Products/AnalysisEdition.shtml and mAdb http://madb.nci.nih.gov/ generating respectively, gene lists A (additional file 1) and B (additional file 2). In both cases, the results were tested for statistical significance, using a classic two-tailed T-test and we set a threshold of at least a two-fold up-regulation for genes to be considered enriched. In gene list A, a correction of Benjamin and Hochberg was applied. In both lists, the results were log_2 _transformed. We found more than 3,400 genes which were enriched by at least two-fold in the GFP + fraction with a p < 0.0001. In gene list B, 1,196 genes were removed for having an insufficient number of values and we found more than 1,180 genes which were enriched by at least two fold in the GFP + fraction with a p < 0.0001. Next we ranked the lists from the highly enriched genes down and manually compared the two lists. To simplify, we concentrated on the 150 top ranked genes, which were genes that were enriched by at least four-fold. We found a remarkable correspondence between the two lists, giving us great confidence about the strength of the analyses. As gene list B was more stringent and more complete regarding gene annotation, we decided to restrict all further analysis to this list. Both full lists A and B can be found as additional files 1 and 2 respectively.

Strikingly, we found in both lists that the top enriched genes were all pancreatic enzymes, which was unexpected. This might be caused by the intrinsic high background fluorescence present in the 5 dpf larvae in the gut, which could contaminate the GFP + cells population during the FAC sorting and would biased all subsequent steps. Alternatively, there are a small number of bona fide GFP + cells in the pancreas (not obvious by normal visual inspection) that are being enriched. In either case, a small number of cells would show very strong enrichment for pancreatic enzymes since there would be essentially no expression of genes such as *elastase *in other tissues.

### Validation of the microarray data

Using published expression data, we validated the gene list by looking for genes already known as been expressed in accessory cells. Our main resource was the zebrafish model organism database (ZFIN, http://zfin.org/cgi-bin/webdriver?MIval=aa-xpatselect.apg). Using "lateral line" as anatomical search term, the database showed 95 expressed genes. However, most of those genes were expressed mostly in the hair cells. When restricting the search to accessory cells (or support cells) only 10 genes were listed. Next when available, we checked the whole mount *in situ *hybridization (WISH) images for each gene, first to confirm the expression pattern and second to see when and where in the embryo this gene was expressed during development. One limitation of our approach was that any gene which was also expressed in other tissues or organs would be masked in our arrays, as we were subtracting the accessory cell's expression against the whole larvae. A good illustration of this was the two following genes, *ClaudinB (cldnB*) and *the epithelial cell adhesion molecule (epcam*, previously known as *tacstd)*. Both of those genes were well-described markers for the lateral line [23,24]. *CldnB*, from early on in development is expressed strongly in the lateral line (both in hair cells and accessory cells) which is one of the only tissues it is expressed in, with the exception of an early signal in the brain and the nephritic ducts. In our list this gene is highly enriched (3.48 fold, p = 5.71E-20). In contrast, *Epcam *started out as a fairly ubiquitously expressed gene that gets progressively restricted to the lateral line, the pharyngeal pouch and the pharyngeal endoderm. This gene was also enriched, but more modestly (1.43 fold, p = 9.75E-13).

In addition we looked at genes, which have been reported in the literature as being expressed in supporting cells of sensory neuroepithelia in various animal models. However, most of those genes were expressed in the supporting cells and also in a number of other structures at various stages. For example, this was the case for the genes of the *notch *family (notch1a, notch3) [10], which were not found as significant enriched in our data set (0.5 fold for both and p = 2.76E-05 and p = 7.36E-08 respectively), as those genes were expressed in the accessory cells, but also in a number of other tissues at all stages. Another well-documented marker of accessory cells in neuromasts, *keratin 15 (krt15) *[25] which is specific to the lateral line from early on with the exception of the pharyngeal arches and the gut, was substantially increased in our list (3.47 fold, p = 2.32E-13). Therefore, we concluded that our approach was valid, as most of the known markers of accessory cells of the lateral line behaved as expected in our data set.

### New markers validated by WISH and qRT-PCR

**Figure 5:**
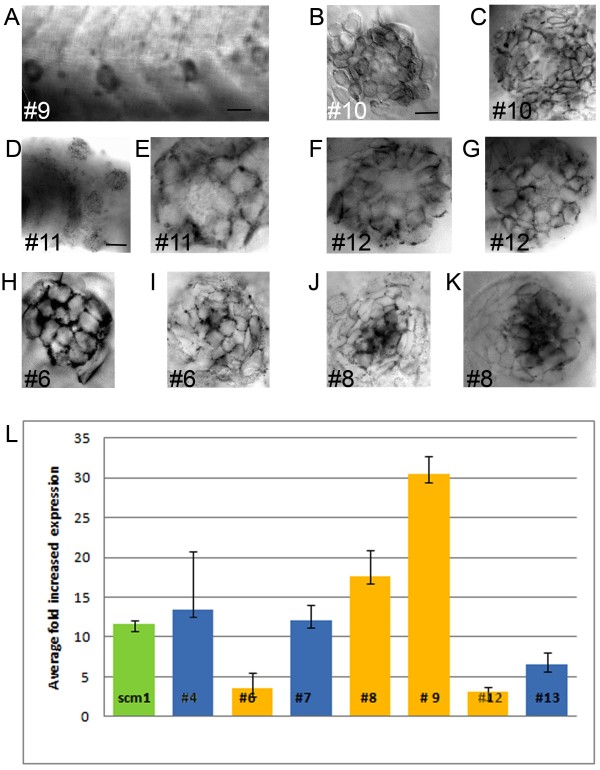
**WISH and q-RT-PCR on novel genes enriched in accessory cells A to K**. Whole mount *in situ *hybridizations (WISH) in 3 and 5 dpf larvae A ring like staining is visible, because hybridization is excluded from centrally located hair cells and only found in peripheral accessory cells for gene #9, 10, 11 and 12 (A to G). In #6 and 8 (H to K), the hybridization is present in the whole neuromast, but appears much stronger in centrally located hair cells. **A**. WISH against #9 in 3 three neuromasts in a 3 dpf larva (A). **B and C**. WISH against #10 in a neuromast in a 3 dpf (B) and a 5 dpf larva (C). **D and E**. WISH against #11 in a portion of the 5 dpf larval head showing 4 neuromasts (D) and of a neuromast in a 3 dpf larva (E). **F and G**. WISH against #12, in two neuromasts in 5 dpf larvae (F and G). **H and I**. WISH against #6 in a neuromast in a 3 dpf (H) and in a 5 dpf larvae (I). **J and K**. WISH against #8 in two neuromasts in 5 dpf larva (J and K). - is 50 microns in A and D, and 5 microns in B. **L**. Average fold increased expression of genes determined by qRT-PCR. The *tnks1bp1 *gene is in green. Genes showing an expression pattern by WISH (in A to Q) are in yellow the others are in blue. Error bars represent standard deviation.

Our main interest was to find new markers for the accessory cells of the lateral line. Based on our primary analysis of the microarray data, we reasoned that genes enriched more than fourfold would be specifically expressed in accessory cells of the olfactory epithelium and/or the lateral line (with failures possible from cell contamination). Therefore, we randomly chose 15 genes (out of the top 150 enriched genes), which had not been previously described as expressed in the lateral line (Table 1, #1 to #15). We assessed them in a qualitative (WISH, Figure 5A to 5K) and a quantitative approach (quantitative qRT-PCR, Figure 5L).

**Table 1:** List of 15 randomly chosen genes tested by WISH and qRT-PCR

#	Gene identifier	Enrichment (fold)	P value	Gene name/symbol	Position in list B
1	BI705588	6.37	8.19E-15	elastase 2 (ela2), mRNA.	19
2	BM101644	5.71	2.25E-19	Similar to Apolipoprotein D	39
3	BM101698	5.35	2.56E-17	Wu:fk35f04	60
4	BI843214	5.11	4.69E-18	Hypothetical LOC571373	71
5	NM001081690	5.22	1.77E-16	Similar to chymotrypsinogen B	64
6	AI497473	4.79	6.70E-13	si:dkey-14d8.6	88
7	BE201597	4.97	1.56E-20	Uncoupling protein 2	78
8	NM001113659	4.57	5.47E-19	zgc:198419	101
9	AW282106	4.55	8.94E-19	Transcribed locus	103
10	AF308598	4.64	2.02E-21	atp1a1a.3	99
11	BM183918	3.96	4.59E-09	Zgc:56136	155
12	AW184433	4.23	7.67E-22	ms4a17a.11	126
13	AW184269	4.30	8.24E-22	si:dkey-127j5.5	122
14	BI842844	4.11	2.01E-08	c1qtnf5	137
15	AB081314	3.98	1.76E-22	nfe2l2	153

Column1: The numbers (#) attributed from 1 to 15 to the randomly chosen genes. Column 2: The gene identifier, as found in NCBI databases. Columns 3 and 4: The enrichment fold and the p-value respectively, as determined with the mAdb analysis of the microarray results. Column 5: The gene's name as described in the NCBI databases. Column 6: Ranking of the gene in list B determined in the mAdb analysis of the microarray results.

First, we made antisense probes for WISH. Six out of the fifteen probes reliably gave us a strong and specific staining in the neuromasts (Figure 5A to 5K). For the rest of the probes we obtained inconclusive results, possibly because of their poor quality, as we sometimes had only very small sequences to choose from (i.e. ESTs). Accessory cells were strongly and exclusively labeled in four out of those six genes/ESTs (9, 10, 11 and 12). This was visualized in a ring like staining absent from the center of the neuromast (Figure 5A to 5G). Gene 9 (accession # AW282106) was documented as a transcribed locus, weakly similar to NP_001136055.1 *immediate early response2 *with an enrichment = 4.55 fold (p = 8.94E-19) (Figure 5A). Gene 10 (accession # AF308598) was described as *atp1a1a.3 ATPase, Na +/K + transporting, alpha 1a.3 polypeptide*, with an enrichment = 4.64 fold (p = 2.02E-21) (Figure 5B and 5C). Gene 11 (accession # NM_200198.1) is heme binding protein 2 (hebp2) and had an enrichment = 3.96 (p = 4.59E-09) (Figure 5D and 5E). Gene 12 (accession #AW184433) with an enrichment = 4.26, (p = 7.67E-22) (Figure 5F and 5G) was described as a member of *the membrane-spanning 4-domains, subfamily A, member 17A.11 *(ms4a17a.11). Finally, the two remaining genes 6 and 8 were staining more strongly the hair cells in addition to the accessory cells. Gene 6 (accession # AI497473) was also poorly described (Si:dkey-14d8.6, Dr.77222) and had an enrichment = 4.79 fold (p = 6.70E-13) (Figure 5H and 5I). Gene #8 (accession # NM_001113659) was documented as similar to *Ferritin heavy chain (Ferritin H subunit*), also called, *Cell proliferation-inducing gene 15 protein*, enrichment = 4.57 fold (p = 5.47E-19) (Figure 5J and 5K). Noticeably, all of the above genes had an expression pattern restricted to neuromasts. Therefore, we concluded that our approach was an effective way of uncovering new markers for the population of interest, namely the accessory cells of the lateral line.

Next we designed primer sets for performing qRT-PCR on the same 15 genes (Table 1) and on the *tnks1bp1 *gene. We obtained results for 7/15. We saw an increased expression ranging between 4 and 30 fold (Figure 5L) in the GFP + cells. Genes that were showing both staining in WISH and gave results in the qRT-PCR were depicted in yellow. Genes with results only in qPCR were shown in blue and the *tnks1bp1 *gene, which was increased by 12 fold, was depicted in green. In conclusion, we confirmed for 4 tested genes (#6, 8, 9 and 12) the enriched expression that was seen by WISH and an additional 3 genes had enriched expression by qRT-PCR. Taken all together, we assessed 15 randomly chosen genes with quantitative and qualitative methods and found a specific expression in the neuromast and/or an increased expression in the GFP + cell population in 9 of them. Thus, assessing randomly chosen genes with quantitative and qualitative methods, we confirmed the validity of the combinatory approach of FACS and microarrays to establish the transcriptome of accessory cells in zebrafish.

## Discussion

Accessory cells of the lateral line organ in zebrafish are known to comprise a subpopulation of progenitor cells, but are still very poorly characterized at the molecular level. We have established and characterized a new transgenic line Tg (*tnks1bp1*:EGFP), which has a restrictive and specific GFP expression in this small population of cells in zebrafish larvae. We have cloned a putative homolog of *tnks1bp1 *with a highly specific expression restricted to accessory cells. We will further pursue the functional analysis of this gene in the accessory cells of the lateral line.

We have then utilized this transgenic line in a combined approach of FACsorting and microarray analysis in order to gain molecular insight into the accessory cells of the neuromasts. The intrinsic limitations of our assay were twofold. As we were subtracting RNA from GFP + cells against RNA from whole larvae, we could only find enrichment in genes, which are not expressed, or expressed at very low levels, in other tissues of the larvae. A second limitation of our approach was the fact that in addition to GFP expression in the lateral line, it was also expressed in the olfactory epithelium. Thus, as a first approach, it is difficult to distinguish genes specific to either neuroepithelia. Only WISH done gene by gene will distinguish between the two organs. However, this will be useful information, as it will allow establishing parallels and differences between two regenerating tissues in the fish. Furthermore, the olfactory epithelium is well known for its regenerative capacity into adulthood, even in mammals. This will allow further comparison with non-regenerating sensory epithelia in higher vertebrates, like the inner ear.

We found the surprising result that the top enriched genes were all pancreatic enzymes. While there was no obvious expression in the pancreas, this possibility is not ruled out, nor is the possibility of contamination in our enrichment because of autofluorescence.

Nevertheless, we could convincingly show enrichment in genes specific to accessory cells of the lateral line. First as expected, we found enrichment in genes that were known to be expressed by accessory cells. Second, we found and described new markers, which were specific to neuromasts and in most cases to accessory cells. Not all genes that we picked for testing by WISH or qPCR gave us results. One plausible explanation is that many of the oligomers chosen for the "in house" microarrays correspond only to short ESTs, which often offer only very little sequence to design good antisense probes or primers for q-PCR. Another possibility is that expression levels are too low to be robustly detected by WISH. This brings up a clear limitation of the microarray approach in general, as you only ever interrogate the limited pool of genes that have been preselected while building the array.

## Conclusions

We present a new transgenic eGFP zebrafish gene trap, which landed in a putative homolog of the *tnks1bp1 *gene. The trapped expression is a highly-specific and restricted to cells, which comprised progenitor cells in the lateral line and olfactory epithelium. The putative function of this gene in maintenance of telomere length [22] would fit perfectly with the characteristics of such a cell population, but remains to be investigated. The transcriptional signature presented here will facilitate other studies aiming at the elucidation of the molecular mechanisms governing the regenerative potential of sensory epithelia.

## Methods

### Fish care and husbandry

Fish care and husbandry were performed according to Westerfield & al [26] in compliance with NIH guideline for animal care.

### Establishment of the *tnks1bp1*:EGFP transgenic line

Injections of the Tol2 EGFP splice accepting gene trap construct T2KSAG was injected exactly as previously described [27].

### Live imaging

Imaging of the lateral line in live larvae was performed using an inverted Zeiss AXIOVERT200M equipped with an Apotome grid confocal system. Larvae were anesthetized with MS222 (0.005%) and mounted on a cover slip in 2% Methylcellulose (Sigma).

### Immunofluorescence of larvae and cryosections

Larvae were fixed o/n with 4% formaldehyde (Electron Microscopy Sciences) in 1 × PBS (Quality Biological, Inc.), at various stages and subsequently stored in 100% methanol. After progressive rehydration (25%, 50% 75% and 100% PTW (PBS1x, 0.001% Tween and 0.001% DMSO)), larvae were treated with acetone for 7 minutes at -20°C. Subsequently, we rehydrated and rinsed them for 3 × 5 minutes in PTW. Next we digested them with 1 mg/ml collagenase (Sigma) in PTB (PTW + 10% goat serum + 10% BSA) for 35 minutes. After 5 × 5 minute rinses with PBT, we pre-incubated the larvae 4 hours in PBT. Larvae were incubated o/n with the polyclonal rabbit primary antibody (1/200) against Myosin VI (Proteus Biosciences, Inc) and a fluorescently labeled monoclonal mouse primary antibody (1/200) against GFP (Abcam). The next day we rinsed the larvae 6 × 10 minutes in PTW and pre-incubated them again for 4 hours in PBT. The fluorescently labeled secondary anti-rabbit antibody (1/500) was added o/n. The next day 6 × 10 minute rinses in PTW were performed. Larvae were mounted on slides in Aquapolymount (Polyscience, Inc) for imaging on an upright confocal microscope (Zeiss AXIOVERT).

Cryosections were prepared by embedding larvae in cryomount plastic cupules (VWR Scientific) in Cryostat sectioning Embedding Compound (VWR Scientific) which was brought to freezing temperature by putting them in a closed container on dry ice, before transferring them to the -80°C. Ten and twenty microns sections were collected by cryostat (Zeiss).

### Embryo dissociation and fluorescence activated cell sorting (FACS)

Tg (*tnks1bp1*:EGFP) larvae transferred to glass dishes were anesthetized on ice. As much water as possible was removed and replaced by a + 0.25% trypsin and 1 mM EDTA solution (GIBCO) and incubated for 15 to 30 minutes at room temperature during which the embryos were dissociated mechanically with a 200 μL pipet tip by pipetting them up and down. The digestion was stopped by adding fetal bovine serum to a 10% final concentration. Cell suspensions were then filtered through 40 μm nylon mesh, washed twice with PBS, pelleted by centrifugation at 600 × *g *for 2 minutes and resuspended in L-15 medium (Sigma) supplemented with 10% fetal bovine serum. FACS of single cell suspensions was performed at room temperature using a FACSAria Sorter (Becton Dickinson) with a Coherent Innova 70 laser at 488 nm and 200 mW power. GFP + and GFP- cells were sorted directly into Trizol Reagent (Invitrogen) and if necessary, stored at -80°C.

### Microarray analysis

We used "in house" printed 33 K zebrafish oligo microarray slides, which consisted of oligo sets derived from three sources: Compugen (with 16,512 × 60 mers), MWG (with 14,240 × 50 mers) and Operon (3,479 × 70 mers). The set contains replicates of several positive (known housekeeping genes) and negative control oligos (random sequences) to control for the homogeneity and specificity of the hybridization. Fifteen biological replicates of RNA extracted from GFP positive cells were co-hybridized with control RNA (= reference) for a total of 20 hybridizations including 5 dye swaps. Hybridizations were performed overnight at 45°C in Maui Mixer FL hybridization chambers (BioMicro Systems). Microarray slide post-hybridization processing and scanning were done as previously described [25]. Data points with average quality values below 1.0 were eliminated and the datasets were normalized by Lowess (R-Bioconductor).

Data analysis to identify differentially expressed genes was carried out using two different software packages. First, we did a pair-wise comparison using GeneSifter http://www.genesifter.net/ with normalized data, which was log_2 _transformed. The GFP + and the reference RNA values were separately averaged over the 20 experiments. Fold differences were calculated from log averages and Student's t-test with Benjamini and Hochberg correction to generate p-values that were used to determined statistical significance. Second, we use the online NCI mAdb microarray data analysis tools http://madb.nci.nih.gov/. A simple t-test analysis was performed with a p-value of < 0.001 and a mean fold change of 2 as cut-off.

### RNA isolation and quantitative RT-PCR

Total RNA was isolated by extraction with Trizol Reagent, according to the manufacturer's instructions. RNA pellets were resuspended in nuclease-free water (Ambion). RNA integrity was confirmed by separation and visualization of the ribosomal RNA in ethidium bromide stained formaldehyde/agarose gels according to standard protocols and on nanochips for the Bioanalyzer (Agilent). Approximately 500 ng to 800 ng RNA was linearly amplified by using the Amino Allyl MessageAmp II aRNA Amplification kit (Ambion) with yields ranging from 12 to 30 μg of aRNA. aRNA samples were split and labeled, half with Cy3 mono NHS ester and half with Cy5 mono NHS ester (CyDyes (GE Healthcare); post-labeling reagents (MessageAmp II kit, Ambion).

Quantitative PCR was performed using SuperScript™ III Platinum^® ^SYBR^® ^Green One-Step qRT-PCR (Invitrogen) using 20 ng of RNA and 5 μM gene specific-primers in a 25 μL reaction, according to the manufacturer's instructions. PCR primers were as follows: α-actin 5'-TGCTGTAACCGAACTCATTGTC and 5'-CAAGCTTACTGGTATGGCCTTC; BI705588 5'- GACACAACTCCATCTCCACC and 5'-CTACCTGCACTAAATCTGACTGG; BM101644 5'- CAGGAATACTCAGCACGGAAG and 5'- CATACTGGGTTCTGGCTACTG; BM101698 5'- GATGAGGTTTGAGTTCGGAGG and 5'-TGGACAGATTCATGCACTCTC; BI843214 5'- GAGCACCAGCATCTAAAACAC and 5'- AAAGGTCACGCAGAAACAAATC; NM_001081690 5'-AACGACAACTTCCCTGGTG and 5'-GATTTTGTTTCCCCAGAAGCG; AI497473 5'-GTGAAGAGCCAACATGAGAATAAG and 5'-CAGTCTGAACCAGAGCTAAAGG; BE201597 5'- CGCCAACTTATTCAGCATGTG and 5'-CAAAGACATGCGCTATTGGG; BC154746; NM_001113659 5'-CCACATCACTAACCTCTCCAAG and 5'-ATTTAGCTGTCCAGGGTGTG; AW282106 5'-AGTTCAACCAGTTCATCCGAG and 5'-CAGAACGAGCAGGATTACAGG; AF308598 5'-TTGAAGCTGTGGAGACTCTTG and 5'-GTCTGGTTCTCTGTGGTATCG; BM183918 5'- GCTTGTGTTTATATGGGCGC and 5'-GAAAATGACCTGTTTCAATCTTTGG; AW184433 5'-GAGACGAGATTCTGCTTCTGG and 5'-GTTGGTGCTGTTTGTGTCG; AW184269 5'-TGCTTTCCTTGGCGGTATAC and 5'-TGTGCCTTCATTTTGGGTTG; BI842844 5'-CAAGATTCCCAGTTTGTGCAC and 5'-TCTCCCCTCTTTCTCCTTTCTC; AB081314 5'-TTCGAGATGAGAACGGAAAGG and 5'-AAGGCGAGGAACTAGGAAAAC. The primers for the red *tkns1bp1 *probe were: 5'- TGC ATT TAC AAA CAT ATG GAGTAATTT TAC and 5'- ATG AAC ATG GTT AGC AGA GGG, for the blue *tkns1bp1 *probe were: 5'- CGA CAG GAC ACA AAC AGA CG and 5'- TCA TCT GGGTTA ATG AGC GC.

### Whole mount *in situ *Hybridization (WISH)

WISH was performed as described previously [28]. We designed antisense probes, using the following primers BI705588 5'-GGAATGTATCCAGACACACGGGTG and 5'-TAATACGACTCACTATAGGGCAAGCACAACCTGCCACTCAGC; BM101644 5'- GCACAGCTCCGGTCTCTGGTCG and 5'-TAATACGACTCACTATAGGGGTGTCTCTGACTCTGCTGGCAG; BM101698 5'-CGCAGTTTGTCCAGCTGGCAGCAG and 5'-TAATACGACTCACTATAGGGCTTTATATCTGAGGAGGCCTGTGG; BI843214 5'-CAGAGGTTGATGACCAGACCTTCAC and 5'-TAATACGACTCACTATAGGGCCGACAGAGACAGCTGTACAGAAATG; NM_001081690 5'-CTGCCATCCCTCCTGTTATTACCGG and 5'-TAATACGACTCACTATAGGGGTAAACACCGGGAGAACTGG; AI497473 5'-GCCAATGGACAAGAGTTACGCTGG and 5'-TAATACGACTCACTATAGGGCAACACCCGTAAGGAAGTGTTGG; BE201597 5'-CGCAGCGAATGCCCTTCCAGC and 5'-TAATACGACTCACTATAGGGGAGGGCAGTCCTTATTGTTGAGCC; BC154746??; NM_001113659 5'-GAACGGAACTTCGATCTCGTCG and 5'-TAATACGACTCACTATAGGGCCACTTTGGAGCATGCAGATG AW282106 5'-CATCACAGTACGTGTTCTGTCTCG and 5'-TAATACGACTCACTATAGGGGGATCACTCCGAGAATTGATCTC; AF308598 5'-GACCCAGAACCGGATGACTGTTGC and 5'-TAATACGACTCACTATAGGGGAAGGACGTCATCCAGCTGTTC; BM183918 5'-GCACCAGATTAAGCTTTCTGAGAC and 5'-TAATACGACTCACTATAGGGCCCCCATTCCCTGGATGTGG; AW184433 5'-CAGCCGAGCCACAGAGTGAAGTC and 5'-TAATACGACTCACTATAGGGGCAATGCCTGCAGCTATAGCACTG; AW184269 5'-CTTGCTCAAATAGGAGACATGC and 5'-TAATACGACTCACTATAGGGGGTGTAAACTCTTGGAAATCGTGTGCC; BI842844 5'-CTGCAGACATGGCCTCTCTCATTG and 5'-TAATACGACTCACTATAGGGCTGCAAGCTGGAGCGGTAGACGG; AB081314 5'-CGATCCCATGTCATTCGATGAGTG and 5'-TAATACGACTCACTATAGGGGAGGTTTGGAGTGTCCGCTAGC. Probes were hybridized at 60°C. Larvae were mounted on coverslip slides in glycerol (Sigma) for imaging on an inverted microscope (Zeiss AXIOVERT).

## Authors' contributions

MB conceived, designed and participated in the acquisition and interpretation of most data and wrote the manuscript. VEG and AT did the WISH and qRT-PCR. AE hybridized the microarrays and helped with the analysis. JB and VEG performed the microarrays analyses. JI helped with the FACS sorting and preparation of RNA samples for the microarrays. JI and SZ did the genomic mapping of the gene-trap. KS and BW constructed and did the gene-trap. CS and SH provided Tg (atoh1a: dTomato) prior to publication. LX performed in situ hybridization and molecular biology support. SB helped with the conception, design and interpretation of data and writing of the manuscript. All authors read and approved the final manuscript.

## Supplementary Material

Additional file 1 **Table S1(= list A). Extensive list of genes significantly enriched, generated with GeneSifter. http://www.geospiza.com/Products/AnalysisEdition.shtml**. We ranked the list from the highly enriched genes down. The gene designation, the p value and the enrichment fold are presented. We found more than 3,400 genes which were enriched by at least two-fold in the GFP + fraction with a p < 0.0001. Click here for file

Additional file 2 **Table S2 (= list B). Extensive list of genes significantly enriched, generated with mAdb. http://madb.nci.nih.gov/**. Next we ranked the lists from the highly enriched genes down. The gene designation, the p value and the enrichment fold are presented. 1,196 genes were removed for having an insufficient number of values and we found more than 1,180 genes which were enriched by at least two fold in the GFP + fraction with a p < 0.0001. Click here for file
